# Safety and efficacy of microinvasive glaucoma surgery with cataract extraction in patients with normal-tension glaucoma

**DOI:** 10.1038/s41598-021-88358-6

**Published:** 2021-04-26

**Authors:** Enchi Kristina Chang, Sanchay Gupta, Marika Chachanidze, Nathan Hall, Ta Chen Chang, David Solá-Del Valle

**Affiliations:** 1 Massachusetts Eye and Ear, Department of Ophthalmology, Harvard Medical School, Boston, MA USA; 2 Bascom Palmer Eye Institute, Miami, FL USA

**Keywords:** Eye diseases, Optic nerve diseases, Outcomes research

## Abstract

This study assesses the safety and efficacy of microinvasive glaucoma surgery (MIGS) with cataract extraction in patients with normal-tension glaucoma (NTG). In our sample of 45 NTG patients, mean intraocular pressure (IOP) decreased from 13.7 to 12.3 mmHg at 2.5 years, and mean medication burden decreased from 2.0 to 1.1 at 1.5 years. For success defined as IOP reduction ≥ 30% from baseline IOP with medication burden reduction from preoperative levels, success probability was 5.4% at 1.5 years. For success defined as medication burden reduction with an IOP reaching goal IOP as determined by the glaucoma specialist, success probabilities were 67.2% at 1.5 years and 29.4% at 2.5 years. At the last follow-up visit, eyes with two MIGS procedures with different mechanisms of action achieved successful medication reduction 68.8% of the time versus 35.7% achieved by a single MIGS procedure (*p* = 0.052). At their last visit, visual acuity was unchanged or improved in all eyes (100%). MIGS with cataract surgery results in modest reductions in IOP and medication burden in NTG patients, which may lead to lower costs and better therapeutic compliance. A combination of two MIGS procedures with different mechanisms of action may potentially be more effective in reducing medication burden than a single MIGS procedure in NTG patients. Further research is necessary to ascertain whether MIGS for NTG patients may help decrease medication burden while helping achieve goal IOP.

## Introduction

Microinvasive glaucoma surgery (MIGS) is increasingly used as an alternative to traditional incisional surgery in the treatment of mild-to-moderate glaucoma. MIGS encompasses a variety of microsurgeries that can be categorized by their mechanisms of action, which include enhancing flow through the trabecular meshwork, shunting aqueous humor to the subconjunctival space, and decreasing aqueous humor production^1,2^. While traditional glaucoma filtering surgeries such as trabeculectomies and tube shunts hold a larger risk of complications such as endophthalmitis, hypotony, bleb leaks, fibrosis, and bleb infections^3–7^, MIGS offers an improved safety profile with relatively few postoperative complications, as the typically smaller incision in MIGS results in a lower risk of incision-related complications^8–13^. MIGS is also often performed in combination with cataract extraction, particularly if patients also have visually-significant cataracts requiring removal, and the IOP-reducing benefits of both surgeries may be additive^1^.

While MIGS has been shown to decrease intraocular pressure and medication burden in primary open-angle glaucoma (POAG) up to 2 years after surgery^14–19^, its efficacy in normal-tension glaucoma (NTG) alone has yet to be established. Only one prior study by Neuhann and Neuhann has demonstrated a 21.1% IOP reduction at 12 months postoperatively in a subgroup of 18 NTG patients treated with iStent inject with phacoemulsification, although this was not maintained at 24 months^19^. Although the evidence for MIGS in NTG patients is currently lacking, given that IOP reduction is the primary mechanism of treatment for both POAG and NTG^2,20,21^, the efficacy of MIGS in reducing IOP in POAG patients suggests that NTG patients may potentially benefit similarly from MIGS. This case series is the first report of a variety of MIGS procedures in NTG patients. We describe our experiences with the safety and efficacy of MIGS combined with cataract extraction in patients with normal-tension glaucoma.

## Methods

### Study design

This is a retrospective study of NTG patients who underwent a MIGS procedure with cataract extraction between April 2016 and November 2019 at Massachusetts Eye and Ear. Eleven different glaucoma fellowship-trained surgeons performed the procedures, and the type of MIGS was at the surgeon’s discretion. Data collection abided by the Declaration of Helsinki and the Health Portability and Accountability Act. Patient medical records were initially identified from financial claims data using International Classification of Disease codes for low-tension glaucoma and Current Procedural Terminology codes for MIGS procedures. All medical records were then reviewed, and patients were included in this study if they met the following criteria: (1) diagnosis of NTG or low-tension glaucoma, defined as a maximum unmedicated IOP ≤ 21 mmHg; (2) concurrent phacoemulsification with MIGS including endoscopic cyclophotocoagulation, iStent and iStent inject (Glaukos, San Clemente, CA), Kahook Dual Blade (New World Medical, Rancho Cucamonga, CA), or Trabectome (MicroSurgical Technology, Redmond, WA, formerly NeoMedix, Tustin, CA); and (3) a minimum follow-up of 6 weeks. Complex phacoemulsification procedures were included in our study. If patients had undergone procedures in both eyes, the first eye was included in our study.

Demographic and preoperative data collected included patient age, gender, glaucoma diagnosis and stage, previous ocular surgeries, IOP, number of glaucoma medications, and best-corrected visual acuity (VA). Glaucoma stages were defined as circumpapillary retinal nerve fiber layer thinning on OCT with Humphrey visual field findings of no abnormalities for mild glaucoma; a single corresponding inferior or superior deficit for moderate glaucoma; or a combination of paracentral or superior and inferior defects for severe glaucoma^22^. Glaucoma stage was defined as indeterminate if visual field testing could not be performed reliably. IOP was measured with Goldmann applanation tonometry. Preoperative IOP, medication burden, and VA were obtained as an average of the values from two consecutive visits prior to the procedure. Postoperative data were collected at the following time points: 1 day, 2 weeks, 6 weeks, 3 months, 6 months, 1 year, 1.5 years, 2 years, and 2.5 years. At each time point, the IOP, number of glaucoma medications, VA, duration of follow-up, subsequent IOP-lowering procedures, and the presence of postoperative complications such as inflammation in the anterior chamber, hypotony, hyphema, corneal edema, or cystoid macular edema (CME) were recorded.

### Surgical procedure

#### Preoperative care and anesthesia

A retrobulbar block was performed by anesthesia with 5 mL of 1% preservative-free lidocaine and 0.375% preservative-free bupivacaine, along with monitored anesthesia care. The patient's operative eye and ocular adnexa were then sterilized with 5% Betadine solution and draped in the usual sterile ophthalmic fashion. A sterile lid speculum was placed in the operative eye.

#### Phacoemulsification

A paracentesis was created, and Trypan blue was injected into the anterior chamber at the surgeon’s discretion to help stain the trabecular meshwork or anterior capsule followed by balanced salt solution (BSS) and viscoelastic. A clear corneal biplanar incision was created with a keratome, and a complete curvilinear capsulorhexis was performed. Following hydrodissection with BSS, nuclear material was phacoemulsified and removed. Viscoelastic was injected into the anterior chamber and capsular bag, and the intraocular lens implant was injected into the capsular bag and rotated into place.

#### Endoscopic cyclophotocoagulation (ECP)

After phacoemulsification, additional cohesive viscoelastic was used to fully open the sulcus. The curved endocyclophotocoagulation probe was inserted into the sulcus, and the ciliary processes were treated with continuous-wave mode with power and number of degrees at the surgeon’s discretion. Power was titrated until shrinking and whitening of the ciliary processes were observed. A second incision was made with a keratome if needed to treat a higher number of degrees.

#### iStent and iStent inject

After phacoemulsification, additional viscoelastic was injected into the anterior chamber. The patient’s head and microscope were rotated 30 degrees to the desired side. A gonioscopy lens was placed onto the cornea with some viscoelastic material in the interface to visualize the angle. The iStent or iStent inject was introduced into the anterior chamber on its holder and inserted into the nasal trabecular meshwork. Two iStents were inserted with iStent inject, and a single iStent was inserted for all other iStents. For all other iStents, check procedures were performed by gently tapping the stent to secure placement. Then, the patient's head was rotated into the primary position, and the irrigation/aspiration tip was used to remove any residual viscoelastic.

#### Kahook dual blade (KDB)

After phacoemulsification, additional viscoelastic was injected into the anterior chamber. The patient’s head and microscope were rotated 30 degrees to the desired side. A gonioscopy lens was placed onto the cornea with some viscoelastic material in the interface to visualize the angle. The Kahook dual blade was introduced into the anterior chamber and passed through the trabecular meshwork in an inside-out fashion until two strips of trabecular meshwork were formed for approximately 3 to 5 clock hours depending on surgeon preference and visualization. Then, the patient's head was rotated into the primary position, and the irrigation/aspiration tip was used to remove any residual viscoelastic.

#### Trabectome

After phacoemulsification, additional viscoelastic was injected into the anterior chamber. The patient’s head and microscope were rotated 30 degrees to the desired side. A gonioscopy lens was placed onto the cornea with some viscoelastic material in the interface to visualize the angle. The Trabectome handpiece was introduced into the anterior chamber, and between 90 to 120 degrees of nasal trabecular meshwork was treated at a power specified by the surgeon.

#### Closing and postoperative care

At the conclusion of the procedure, Miochol and intracameral cefuroxime were injected into the anterior chamber. If the patient had an allergy to penicillin, intracameral moxifloxacin was given instead. The main wound and the paracentesis wounds were hydrated and found to be free of any leaks. A drop of prednisolone acetate 1% and moxifloxacin 0.5% or Maxitrol ointment were placed on the eye before it was patched and shielded. On postoperative day 1, patients were started on topical prednisolone acetate 1% with ketorolac 0.5% added or loteprednol 0.5% substituted at the surgeon’s discretion depending on the amount of inflammation present. Postoperative drops were tapered at the surgeon’s discretion, and glaucoma medications were added depending on IOP. If IOP were above the goal IOP, then a glaucoma medication was added at that visit.

### Outcome measures

Primary outcome measures at each postoperative visit were IOP reduction, glaucoma medication burden, visual acuity, cumulative success probabilities from Kaplan–Meier analyses, and complication rates. For survival analysis, two different success criteria were used and defined as follows: postoperative medication burden less than preoperative levels and (Criteria 1) IOP reduction ≥ 30% from baseline medicated IOP; or (Criteria 2) IOP ≤ goal IOP (determined to be ≥ 30% reduction from where the glaucoma specialist noted progression or the IOP at which the glaucoma specialist thought that the patient should be to prevent further progression based on clinical presentation). A failure was recorded if patients did not meet the specified success criteria on two consecutive follow-up visits after 6 weeks, with the latter of the two dates as the failure date. A failure was also recorded if a patient underwent an additional glaucoma procedure or achieved no light perception vision. Kaplan–Meier survival was modeled from 6 weeks onwards to eliminate failures that could result from short-term postoperative fluctuations in IOP. Patients with no preoperative medication burden were excluded from Kaplan–Meier analyses given that medication burden reduction was required for success.

### Statistical analysis

Statistical analyses were performed using R (version 3.6.3). Statistical significance was defined as p < 0.05. Average and standard deviation were calculated for IOP, medication burden, and VA. Line graphs of average values were generated with error bars representing standard error of the mean. Comparisons with preoperative values were conducted with Wilcoxon paired signed-rank tests. Kaplan–Meier analyses were used to generate the average and standard error of cumulative success probabilities, with success criteria defined above. Comparisons of the numbers of successes and failures by type of MIGS procedure were performed with Fisher tests. Hazard ratios for preoperative and demographic characteristics were obtained from Cox proportional hazard regression analyses. Snellen visual acuities were converted to logarithm of minimum angle of resolution (LogMAR) equivalents, with values of 2 and 3 representing count fingers and hand motion vision respectively^23^.

### Ethics approval and consent to participate

This retrospective chart review study was approved for exempt status by the Mass General Brigham Institutional Review Board and abided by the tenets of the Declaration of Helsinki. Informed consent was not required for participation.

## Results

A total of 45 eyes of 45 patients met the inclusion criteria for our study. Patient demographic and preoperative data are summarized in Table 1. All eyes had a diagnosis of NTG. The average follow-up period was 24.1 ± 14.0 months (mean ± SD, range 4.1 – 58.7). Mean preoperative IOP was 13.7 ± 2.2 mmHg (range 10 – 18 mmHg) with a medication burden of 2.0 ± 1.1 (range 0 – 4).

**Table 1:** Demographic and preoperative data.

Parameters		Parameters	
*Demographics*		*Prior Ocular Surgeries, N (%)*	
Eyes	45	None	44 (97.8)
Female Eyes, N (%)	30 (66.7)	RD repair	1 (2.2)
Age (years)			
Mean ± SD	74.4 ± 6.7	*Type of Procedure, N (%)*	
Range	62 – 94	Phaco/ECP	2 (4.4)
Race/Ethnicity, N (%)		Phaco/ECP/iStent G1^b^	7 (15.6)
White	32 (71.1)	Phaco/ECP/iStent G2^c^	1 (2.2)
Black or African-American	9 (20.0)	Phaco/ECP/KDB^d^	8 (17.8)
Asian	4 (8.9)	Phaco/iStent G1^b^	12 (26.7)
		Phaco/KDB^d^	5 (11.1)
*Glaucoma Stage, N (%)*		Phaco/Trabectome^e^	10 (22.2)
Indeterminate	1 (2.2)		
Mild	19 (42.2)	*Preoperative Baseline*	
Moderate	22 (48.9)	IOP (mmHg)	
Severe	3 (6.7)	Mean ± SD	13.7 ± 2.2
		Range	10 – 18
*Prior Glaucoma Laser, N (%)*		# of Glaucoma Medications	
None	29 (64.4)	Mean ± SD	2.0 ± 1.1
ALT	2 (4.4)	Range	0 – 4
LPI	2 (4.4)	Visual Acuity	
LTP^a^	1 (2.2)	Mean ± SD	0.25 ± 0.20
SLT	10 (22.2)	Range	0 – 0.88

N = number of eyes; SD = standard deviation; IOP = intraocular pressure; mmHg = millimeters of mercury; Phaco = phacoemulsification; ALT = argon laser trabeculoplasty; LPI = laser peripheral iridotomy; LTP = laser trabeculoplasty; SLT = selective laser trabeculoplasty; RD = retinal detachment; ECP = endoscopic cyclophotocoagulation; G1 = Generation 1; G2 = Generation 2; KDB = Kahook Dual Blade.

^a^This patient could not recall the type of LTP performed, and no records were available from then.

^b^iStent Trabecular Micro-Bypass Stent (Models GTS100R and GTS100L, Glaukos Corporation, San Clemente, California).

^c^iStent *inject* Trabecular Micro-Bypass System (Model G2-M-IS, Glaukos Corporation, San Clemente, California).

^d^Kahook Dual Blade (New World Medical, Rancho Cucamonga, CA).

^e^Trabectome (MicroSurgical Technology, Redmond, WA, formerly NeoMedix, Tustin, CA).

The number of eyes for each procedure is listed in Table 1. Thirteen patients underwent surgery with KDB, and KDB clock hours ranged from 3 to 5 with a median of 4. Eighteen patients underwent endoscopic cyclophotocoagulation with a range of 180 to 330 degrees and average power of 0.324 ± 0.060 Watts (range 0.12–0.4). A second incision was created to treat 330 degrees in a single patient. Trabectome surgery was performed in ten patients with a range of 90 to 120 degrees and mean of 98.9 degrees. Two patients underwent surgery with iStent inject and received two iStents. Otherwise, all other patients with iStent received a single stent.

**Figure 1:**
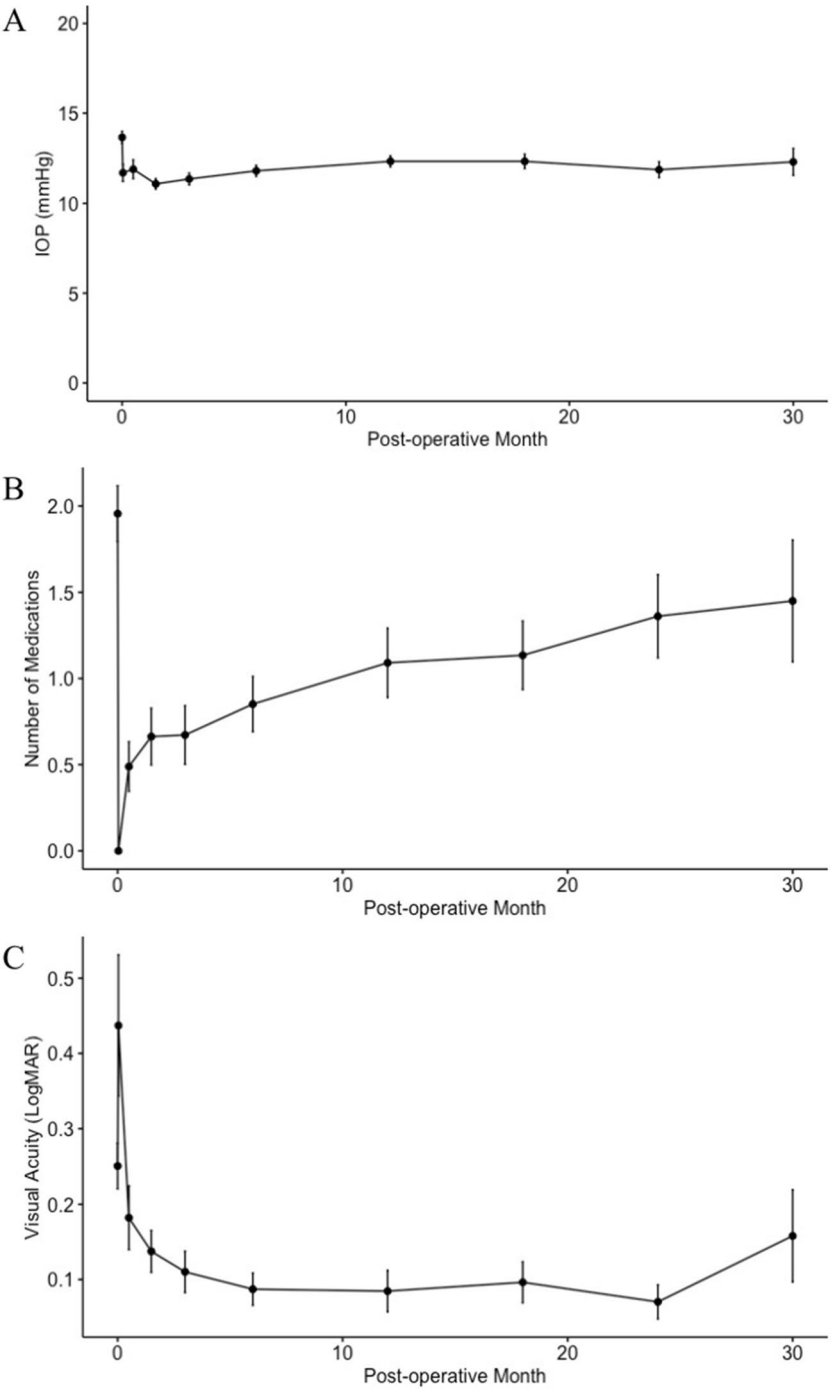
Line graphs of average values of postoperative (**A**) intraocular pressure, (**B**) number of medications, and (**C**) visual acuity over time. Error bars denote standard deviation.

Postoperative intraocular pressure, medication burden, and visual acuity outcomes are summarized in Table 2. Line graphs of postoperative outcomes are shown in Fig. 1. Postoperative IOP was significantly decreased at all follow-up time points compared to preoperative levels. Average IOP reduction at 2.5 years from preoperative levels was 2.1 ± 2.8 mmHg. Medication burden was significantly decreased at all time points up to 1.5 years postoperatively, with an average decrease of 1.1 ± 1.1 medications at 1.5 years. Visual acuity was improved up to 2 years (p = 0.002) and was the same or improved at the last follow-up visit for all eyes (100%).

**Table 2:** Postoperative outcomes data.

	IOP (mmHg)	Medications	VA (LogMAR)
*Preoperative (n* = *45)*			
Mean (SD)	13.7 (2.2)	2.0 (1.1)	0.25 (0.20)
*1 day (n* = *45)*			
Mean (SD)	11.7 (3.3)	0.0 (0.0)	0.44 (0.63)
Decrease from baseline	2.0 (3.2)	2.0 (1.0)	− 0.17 (0.66)
*p* value	< 0.001*	< 0.001*	0.102
*2 weeks (n* = *45)*			
Mean (SD)	11.9 (3.6)	0.5 (1.0)	0.18 (0.28)
Decrease from baseline	1.8 (3.7)	1.5 (1.1)	0.06 (0.30)
*p* value	< 0.001*	< 0.001*	0.012*
*6 weeks (n* = *43)*			
Mean (SD)	11.1 (1.9)	0.7 (1.1)	0.14 (0.18)
Decrease from baseline	2.6 (2.5)	1.4 (0.9)	0.12 (0.19)
*p* value	< 0.001*	< 0.001*	< 0.001*
*3 months (n* = *32)*			
Mean (SD)	11.3 (1.9)	0.7 (1.0)	0.11 (0.16)
Decrease from baseline	2.5 (2.3)	1.3 (0.9)	0.13 (0.18)
*p* value	< 0.001*	< 0.001*	< 0.001*
*6 months (n* = *37)*			
Mean (SD)	11.8 (1.9)	0.9 (1.0)	0.09 (0.13)
Decrease from baseline	1.9 (2.6)	1.2 (0.9)	0.14 (0.17)
*p* value	< 0.001*	< 0.001*	< 0.001*
*1 year (n* = *33)*			
Mean (SD)	12.3 (1.8)	1.1 (1.2)	0.08 (0.16)
Decrease from baseline	1.5 (2.3)	1.1 (1.1)	0.14 (0.25)
*p* value	< 0.001*	< 0.001*	< 0.001*
*1.5 years (n* = *26)*			
Mean (SD)	12.3 (2.1)	1.1 (1.0)	0.10 (0.14)
Decrease from baseline	1.3 (3.0)	1.1 (1.1)	0.11 (0.17)
*p* value	0.019*	< 0.001*	0.015*
*2 years (n* = *18)*			
Mean (SD)	11.9 (1.9)	1.4 (1.0)	0.07 (0.10)
Decrease from baseline	1.7 (3.2)	0.8 (1.2)	0.10 (0.23)
*p* value	0.039*	0.061	0.002*
*2.5 years (n* = *10)*			
Mean (SD)	12.3 (2.4)	1.5 (1.1)	0.16 (0.19)
Decrease from baseline	2.1 (2.8)	0.4 (1.2)	0.10 (0.17)
*p* value	0.041*	0.558	0.122

n = total number of patients at specific follow-up time; SD = standard deviation; IOP = intraocular pressure; mmHg = millimeters of mercury; VA = visual acuity; LogMAR = logarithm of the minimum angle of resolution.

**Figure 2:**
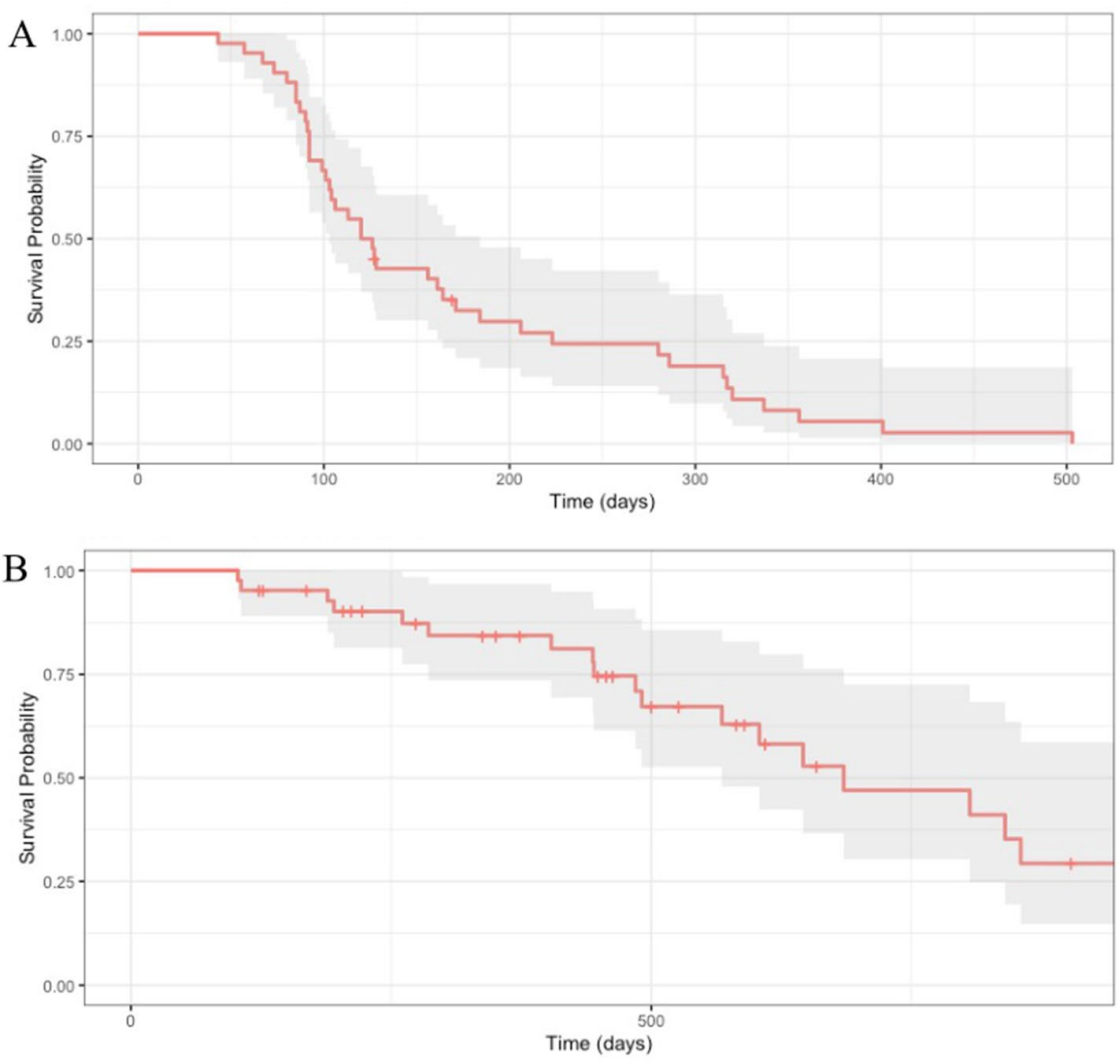
Kaplan–Meier survival curves of microinvasive glaucoma surgery combined with cataract extraction in patients with normal-tension glaucoma. Success criteria were defined as the following: postoperative medication burden less than preoperative medication burden AND (**A**) postoperative intraocular pressure (IOP) reduction ≥ 30% from baseline IOP; or (**B**) IOP ≤ goal IOP. Goal IOP was determined to be ≥ 30% reduction from where the glaucoma specialist noted progression or the IOP at which the glaucoma specialist thought that the patient should be to prevent further progression based on clinical presentation. A failure was recorded if a patient failed to meet success criteria on at least two consecutive follow-up visits, required additional glaucoma procedures, or developed no light perception vision. Patients with no preoperative medication burden were excluded.

Cumulative success probabilities derived from Kaplan–Meier analyses for both success criteria are shown in Table 3, with corresponding Kaplan–Meier curves depicted in Fig. 2. Three patients were excluded from survival analyses given that they were on no glaucoma medications at baseline. For success defined as IOP reduction ≥ 30% from baseline IOP with medication burden reduction (Criteria 1), success probabilities were 32.5% at 6 months and 5.4% at 1 year. For success defined as medication burden reduction with IOP reaching goal IOP (Criteria 2), success probabilities were 67.2% at 1.5 years and 29.4% at 2.5 years.

**Table 3:** Life table.

	Cumulative Success (%) ± SE (95% Confidence Interval)	
	Postoperative < Preoperative # of Medications* AND	
	IOP ≥ 30% reduction from baseline IOP	IOP ≤ goal IOP^b^
6 weeks	100 ± 0.0	100 ± 0.0
	(100, 100)	(100, 100)
N	42	42
3 months	78.6 ± 6.3	100 ± 0.0
	(67.1, 92.0)	(100, 100)
N	42	42
6 months	32.5 ± 7.3	95.2 ± 3.3
	(20.9, 50.6)	(89.0, 100.0)
N	31	39
1 year	5.4 ± 3.7	84.4 ± 5.9
	(1.4, 20.7)	(73.6, 96.8)
N	12	31
1.5 years	-	67.2 ± 8.4
		(52.6, 85.7)
N		21
2 years	-	47.0 ± 10.4
		(30.4, 72.5)
N		12
2.5 years	-	29.4 ± 10.3
		(14.7, 58.5)
N		7

SE = standard error of the mean; N = number of eyes; IOP = intraocular pressure.

*Patients with no medications preoperatively were excluded from this analysis.

^b^Goal IOP was determined by the fellowship-trained glaucoma specialist based on IOP and glaucoma progression.

The demographic characteristics and preoperative baseline data were not significantly different between eyes that received 1 versus 2 MIGS procedures (Table 4). Excluding eyes on no medications at baseline, eleven out of sixteen eyes (68.8%) that received two MIGS procedures achieved successful medication burden reduction by their last follow-up visit, compared to 10 out of 27 eyes (35.7%) that underwent a single MIGS procedure (p = 0.052, Fisher Test). Average IOP was significantly higher for eyes with 1 MIGS compared to eyes with 2 MIGS procedures at 6 months only (Table 5). Visual acuity was significantly worse in eyes with 2 MIGS procedures compared to 1 MIGS procedure at postoperative day 1 only (Table 5).

**Table 4:** Demographic characteristics and preoperative data for eyes stratified by 1 or 2 MIGS procedures.

Parameters	Number of MIGS Procedures		*p* value
	1	2	
*Demographics*			
Eyes	29	16	
Female Eyes, N (%)	19 (65.5)	11 (68.8)	
Age (years)			0.254
Mean ± SD	75.4 ± 6.9	72.5 ± 5.9	
Range	63 – 94	62 – 82	
*Glaucoma Stage, N (%)*			0.604
Mild	11 (37.9)	8 (50.0)	
Moderate	14 (48.3)	8 (50.0)	
Severe	3 (10.3)	0 (0.0)	
Indeterminate	1 (3.4)	0 (0.0)	
*Prior Ocular Surgeries, N (%)*			0.356
None	29 (100.0)	15 (93.8)	
RD repair	0 (0.0)	1 (6.3)	
*Prior Glaucoma Laser, N (%)*			0.632
None	20 (69.0)	11 (68.8)	
ALT	1 (3.4)	1 (6.3)	
LPI	2 (6.9)	0 (0.0)	
LTP^a^	0 (0.0)	1 (6.3)	
SLT	6 (20.7)	4 (25.0)	
*Type of Procedure, N (%)*			< 0.001*
Phaco/ECP	2 (6.9)	–	
Phaco/ECP/iStent G1^b^	–	7 (43.8)	
Phaco/ECP/iStent G2^c^	–	1 (6.3)	
Phaco/ECP/KDB^d^	–	8 (50.0)	
Phaco/iStent G1^b^	12 (41.4)	–	
Phaco/KDB^d^	5 (17.2)	–	
Phaco/Trabectome^e^	10 (34.5)	–	
*Preoperative Baseline*			
IOP (mmHg)			0.418
Mean ± SD	13.8 ± 2.1	13.4 ± 2.5	
Range	10 – 18	10 – 18	
# of Glaucoma Medications			0.446
Mean ± SD	2.0 ± 1.0	1.8 ± 1.3	
Range	0 – 4	0 – 4	
Visual Acuity (LogMAR)			0.546
Mean ± SD	0.27 ± 0.21	0.22 ± 0.19	
Range	0 – 0.88	0 – 0.70	
*Follow-up Length*			0.004*
Mean ± SD	28.0 ± 14.4	17.0 ± 10.0	
Range	4.1 – 58.7	4.2 – 46.1	

N = number of eyes; SD = standard deviation; IOP = intraocular pressure; ALT = argon laser trabeculoplasty; LPI = laser peripheral iridotomy; LTP = laser trabeculoplasty; SLT = selective laser trabeculoplasty; RD = retinal detachment; ECP = endoscopic cyclophotocoagulation; G1 = Generation 1; G2 = Generation 2; KDB = Kahook Dual Blade.

^a^This patient could not recall the type of LTP performed, and no records were available from then.

^b^iStent Trabecular Micro-Bypass Stent (Models GTS100R and GTS100L, Glaukos Corporation, San Clemente, California).

^c^iStent *inject®* Trabecular Micro-Bypass System (Model G2-M-IS, Glaukos Corporation, San Clemente, California).

^d^Kahook Dual Blade (New World Medical, Rancho Cucamonga, CA).

^e^Trabectome (MicroSurgical Technology, Redmond, WA, formerly NeoMedix, Tustin, CA).

**Table 5:** Intraocular pressure, medication burden, and visual acuity outcomes data stratified by 1 or 2 MIGS procedures.

	Number of MIGS Procedures		*p* value
	1	2	
*Preoperative*			
IOP (mmHg)	13.8 ± 2.1	13.4 ± 2.5	0.418
Glaucoma medications	2.0 ± 1.0	1.8 ± 1.3	0.446
Visual acuity (LogMAR)	0.27 ± 0.21	0.22 ± 0.19	0.546
N	29	16	
*1 day*			
IOP (mmHg)	12.1 ± 3.7	10.9 ± 2.2	0.376
Visual acuity (LogMAR)	0.32 ± 0.55	0.64 ± 0.72	0.026*
N	29	16	
*2 weeks*			
IOP (mmHg)	12.1 ± 3.6	11.6 ± 3.6	0.694
Glaucoma medications	0.6 ± 1.1	0.3 ± 0.6	0.314
Visual acuity (LogMAR)	0.13 ± 0.21	0.28 ± 0.37	0.170
N	29	16	
*6 weeks*			
IOP (mmHg)	11.3 ± 2.1	10.6 ± 1.5	0.397
Glaucoma medications	0.7 ± 1.2	0.6 ± 1.0	0.976
Visual acuity (LogMAR)	0.13 ± 0.18	0.15 ± 0.20	0.917
N	27	16	
*3 months*			
IOP (mmHg)	11.5 ± 2.1	11.1 ± 1.5	0.406
Glaucoma medications	0.8 ± 1.0	0.4 ± 0.8	0.111
Visual acuity (LogMAR)	0.12 ± 0.18	0.10 ± 0.12	0.982
N	21	11	
*6 months*			
IOP (mmHg)	12.2 ± 1.8	11.3 ± 1.9	0.030*
Glaucoma medications	0.9 ± 1.0	0.7 ± 1.0	0.509
Visual acuity (LogMAR)	0.07 ± 0.13	0.12 ± 0.13	0.136
N	22	15	
*1 year*			
IOP (mmHg)	12.5 ± 1.8	11.8 ± 1.7	0.319
Glaucoma medications	1.1 ± 1.1	1.2 ± 1.4	0.916
Visual acuity (LogMAR)	0.10 ± 0.18	0.03 ± 0.06	0.206
N	24	9	
*1.5 years*			
IOP (mmHg)	12.3 ± 2.1	12.5 ± 2.0	1.000
Glaucoma medications	1.1 ± 1.1	1.2 ± 0.8	0.653
Visual acuity (LogMAR)	0.10 ± 0.16	0.08 ± 0.08	0.806
N	19	7	
*2 years*			
IOP (mmHg)	11.8 ± 1.8	12.0 ± 2.6	0.807
Glaucoma medications	1.4 ± 1.0	1.0 ± 1.0	0.536
Visual acuity (LogMAR)	0.06 ± 0.10	0.12 ± 0.10	0.306
N	15	3	
*2.5 years*			
IOP (mmHg)	12.7 ± 2.2	-	
Glaucoma medications	1.5 ± 1.2		
Visual acuity (LogMAR)	0.16 ± 0.20		
N	9		

IOP = intraocular pressure; mmHg = millimeters of mercury; LogMAR = logarithm of the Minimum Angle of Resolution; N = number of eyes; SD = standard deviation.

^a^Patients with LP or NLP at these time points were excluded in mean and p value calculations due to a lack of a validated LogMAR equivalent.

Hazard ratios for age, sex, race, glaucoma stage, family history of glaucoma, type of procedure, number of MIGS procedures, baseline IOP, and baseline medication burden were not significant for any of the two success criteria.

Complication rates are shown in Table 6. No complications were seen in any patients after 3 months. Only 1 patient required additional glaucoma surgery 27.8 months after the combined MIGS procedure. This patient had cataract extraction with Trabectome surgery initially and subsequently underwent a trabeculectomy with Ex-PRESS shunt placement due to worsening of visual field deficits and medication intolerance.

**Table 6:** Complication Rates.

		n (%)				
	N	AC Inflammation	Hypotony	Hyphema	Corneal Edema	Cystoid Macular Edema
*Total*	45	33 (73.3)	0 (0.0)	2 (4.4)	21 (46.7)	0 (0.0)
*Early*^1^	45	33 (73.3)	0 (0.0)	2 (4.4)	21 (46.7)	0 (0.0)
*Late*^2^	43	0 (0.0)	0 (0.0)	0 (0.0)	0 (0.0)	0 (0.0)

N = number of patients; n = total number of patients at specific follow-up time; AC = anterior chamber.

^1^Complications present up to 3 months postoperatively.

^2^Complications present after 3 months postoperatively.

## Discussion

The results of this study demonstrate that MIGS with cataract extraction modestly reduces IOP with a positive impact on medication burden. Although postoperative IOP was significantly lower at all follow-up time points compared to preoperative IOP, the magnitude of this reduction rarely achieved the 30% reduction necessary to be considered a success for our Kaplan–Meier survival analysis (Criteria 1). Given that most MIGS procedures rarely achieve an IOP reduction of 30%, Criteria 1 is perhaps unduly stringent particularly as our study includes patients on glaucoma medications at baseline. However, it reflects the magnitude of IOP-reduction suggested in the Collaborative Normal-Tension Glaucoma Study^25^, and the inclusion of Criteria 1 as failure criteria allows for comparison of the present study to this historic, landmark study on NTG. Furthermore, the Kaplan–Meier survival curves looked similar when a 20% cutoff was used (see Supplemental figures).

Although Neuhann and Neuhann found a higher IOP reduction of 3.6 mmHg at 1 year and 2.4 mmHg at 2 years in their NTG cohort undergoing iStent injection with phacoemulsification, their average preoperative IOP of 17.1 mmHg was higher than the preoperative IOP in our study^19^. Prior studies have demonstrated that eyes with a higher preoperative IOP are more likely to experience a larger magnitude of IOP reduction from glaucoma surgery^24,27–30^. Thus, the discrepancy in IOP-reducing efficacy between our study and the Neuhann and Neuhann study could be attributed to the differences in preoperative IOP.

Additionally, we found a significant reduction in medication burden from baseline levels up to 1.5 years postoperatively, with a 67.2% probability of achieving this medication reduction while maintaining a prespecified goal IOP (Criteria 2). While the clinical significance of a 0.9 medication reduction is uncertain, medication cost and number of medications can both be major barriers to therapy adherence^31,32^. Thus, this modest reduction may result in better therapeutic compliance in some patients and may allow a small portion to remain medication free for an extended period of time following surgery.

Furthermore, a larger proportion of patients (11/16) who underwent two MIGS procedures with different mechanisms of action had reduced medication burden at their last follow-up visit after surgery, when compared to patients who received a single MIGS procedure only (10/27), which strongly trended towards significance (p = 0.052). In our cohort, eight patients received ECP with iStent surgery, and another eight received ECP with KDB surgery. It may be possible that combining two procedures with different mechanisms of action may achieve better surgical outcomes in NTG patients, and future studies with larger numbers of patients will be necessary to further elucidate this.

Compared to traditional glaucoma filtration surgeries, MIGS does not achieve as large of a reduction in IOP or medication burden. However, given the minimal postoperative complications from MIGS, there may be a benefit to using MIGS as a bridge to traditional surgeries. A study of trabeculectomy in 17 NTG eyes in Japan demonstrated an IOP reduction from 13.9 ± 0.9 mmHg to 8.1 ± 2.9 mmHg at 5 years with a decrease in medication burden from 3.0 to 0.8 medications^26^. While this decrease is greater than that of our study, significant complications were observed with transient hypotony in 52.9% (9/17), hypotony maculopathy and VA decline ≥ 0.1 unit in 17.6% (3/17), and hyphema and choroidal detachment in 23.5% (4/17) of eyes^26^. Glaucoma drainage implants such as Ahmed and Baerveldt implants may potentially be helpful in some NTG patients, although this has not been explored previously. Although traditional glaucoma filtration surgeries may achieve greater long-term efficacy, MIGS may be considered as a bridge to such surgeries and spare some patients the possibility of long-lasting surgical complications.

Considering that cataract extraction was performed in combination with MIGS in this study, the effects of cataract extraction on our outcomes must be taken into account. First of all, the improvements in visual acuity are most likely attributed to cataract removal, for significant vision improvements would not be expected from glaucoma surgery. In a study by Majstruk et al. of phacoemulsification in medically-controlled POAG, the average preoperative IOP was 17.0 ± 2.7 mmHg with an average reduction of 1.15 mmHg at 1 year, and no significant differences in medication burden were observed at any time point^30^. An even smaller IOP-reducing effect of phacoemulsification could potentially be seen in NTG patients, given the lower preoperative IOP of NTG patients. In another study by Shoji et al., 35 eyes with medically-controlled NTG underwent phacoemulsification alone with an IOP decrease from 16.7 to 15.9 mmHg (p = 0.195) at 1.5 years and 15.8 mmHg at 2.5 years (p = 0.082) with no change in medication burden at either time point^33^. Thus, assuming that cataract extraction does not affect medication burden as seen in their study, the medication burden reduction seen in our study can likely be attributed to the MIGS procedures.

With regards to complication rates, our study demonstrated minimal short-term complications prior to 3 months postoperatively and no long-term complications. No complications were observed in any patients after 3 months. Similar to the minimal risks reported in prior studies of MIGS^8–12^, our study thus demonstrates that MIGS is safe in NTG patients.

The limitations of this study include its retrospective design, small sample size, number of glaucoma surgeons, lack of follow-up data for some patients, and lack of long-term preoperative data due to limitations of our electronic medical record system. Namely, data on later postoperative visits had not been acquired yet for patients who had undergone surgeries more recently, particularly as multiple follow-up visits were displaced due to COVID-19. Thus, the smaller sample sizes at postoperative visits could affect the significance of statistical testing. We also assumed that goal IOP for each patient was determined by the fellowship-trained glaucoma specialists based on whether they thought there was progression or not prior to surgery; however, as many patients have been followed elsewhere or prior to the adoption of our current electronic medical record system, we unfortunately could not determine the exact point at which progression occurred to use as an alternative baseline. Additionally, a comparison group of NTG patients who had undergone cataract extraction alone could not be found given that most NTG patients with progression or on medication who required cataract extraction also underwent an additional IOP-lowering procedure at the time of surgery. Given the small sample size for each MIGS procedure independently and the non-significant hazard ratio for type of procedure for any of our success criteria, subgroup analyses by procedures were not conducted in this study. Thus, our results do not reflect the efficacy of individual procedures, which may potentially vary from our averages.

## Conclusions

In summary, our study results suggest that MIGS with cataract extraction can be used safely and effectively in NTG patients to modestly reduce IOP and medication burden, which may have a positive impact on therapeutic adherence. Performing two MIGS procedures of different mechanisms of action together may potentially be more effective than a single MIGS procedure. Similar to other studies of MIGS, no long-lasting complications or decreases in visual acuity were observed in our study. Unsurprisingly, MIGS did not achieve a 30% IOP reduction in the majority of patients. Further studies may be indicated to expand our sample size over a longer period of time and to explore the efficacy of each MIGS procedure independently and without cataract extraction in NTG patients. Similarly, the potential benefit of two MIGS procedures over a single procedure should be explored further with a larger cohort of patients and in all types of glaucoma.

## Supplementary Information

Supplementary Information

## Data Availability

The data that support the findings of this study are available on request from the corresponding author DS. The data are not publicly available due to them containing information that could compromise research participant privacy.

## Abbreviations

MIGS: Microinvasive glaucoma surgery
NTG: Normal-tension glaucoma
IOP: Intraocular pressure
POAG: Primary open-angle glaucoma
OCT: Optical coherence tomography
VA: Best-corrected visual acuity
CME: Cystoid macular edema
BSS: Balanced salt solution
ECP: Endoscopic cyclophotocoagulation
KDB: Kahook Dual Blade
LogMAR: Logarithm of minimum angle of resolution
SD: Standard deviation
