# Supplementary material for: Safety and efficacy of microinvasive glaucoma surgery with cataract extraction in patients with normal-tension glaucoma
Source: Sci Rep. 2021 Apr 26;11:8910. doi: 10.1038/s41598-021-88358-6 (PMC8076176; doi:10.1038/s41598-021-88358-6)
Supplement: Supplementary file 1 — Supplementary Information [file 41598_2021_88358_MOESM1_ESM.pdf]

A

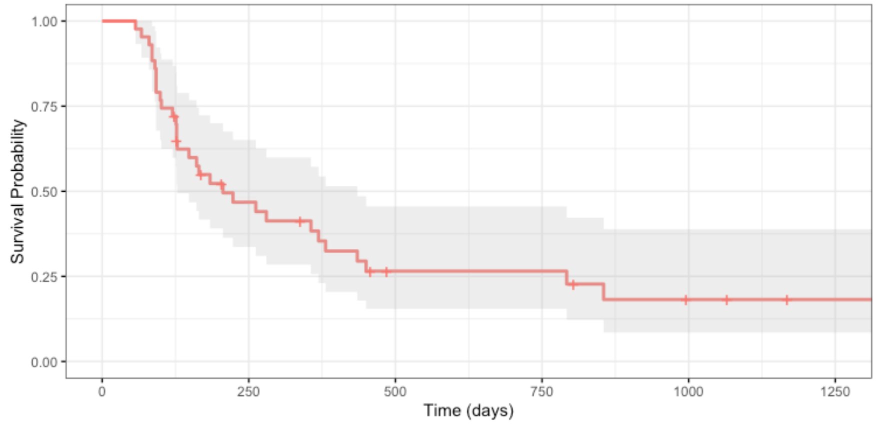

B

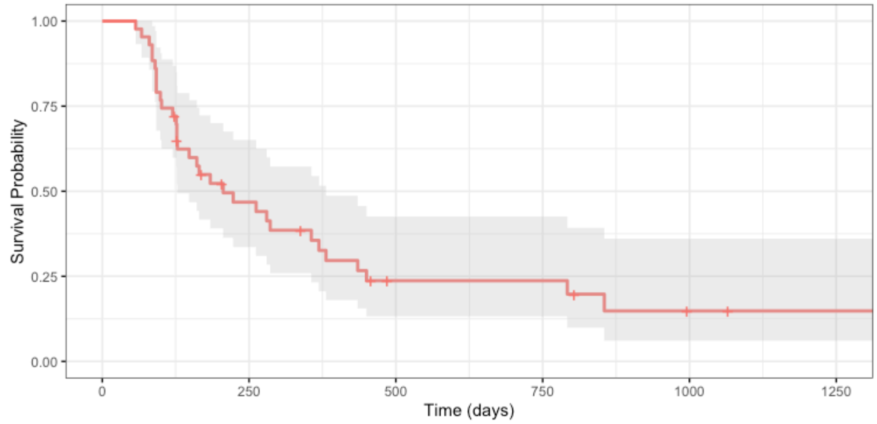

Supplemental Figure 1: Kaplan-Meier survival curves of microinvasive glaucoma surgery combined with cataract extraction in patients with normal-tension glaucoma. Success criteria were defined as the following: (A) postoperative intraocular pressure (IOP) reduction ≥ 20%; or (B) postoperative medication burden less than preoperative medication burden AND postoperative IOP reduction ≥ 20% from baseline IOP. A failure was recorded if a patient failed to meet success criteria on at least two consecutive follow-up visits, required additional glaucoma procedures, or developed no light perception vision. Patients with no preoperative medication burden were excluded.

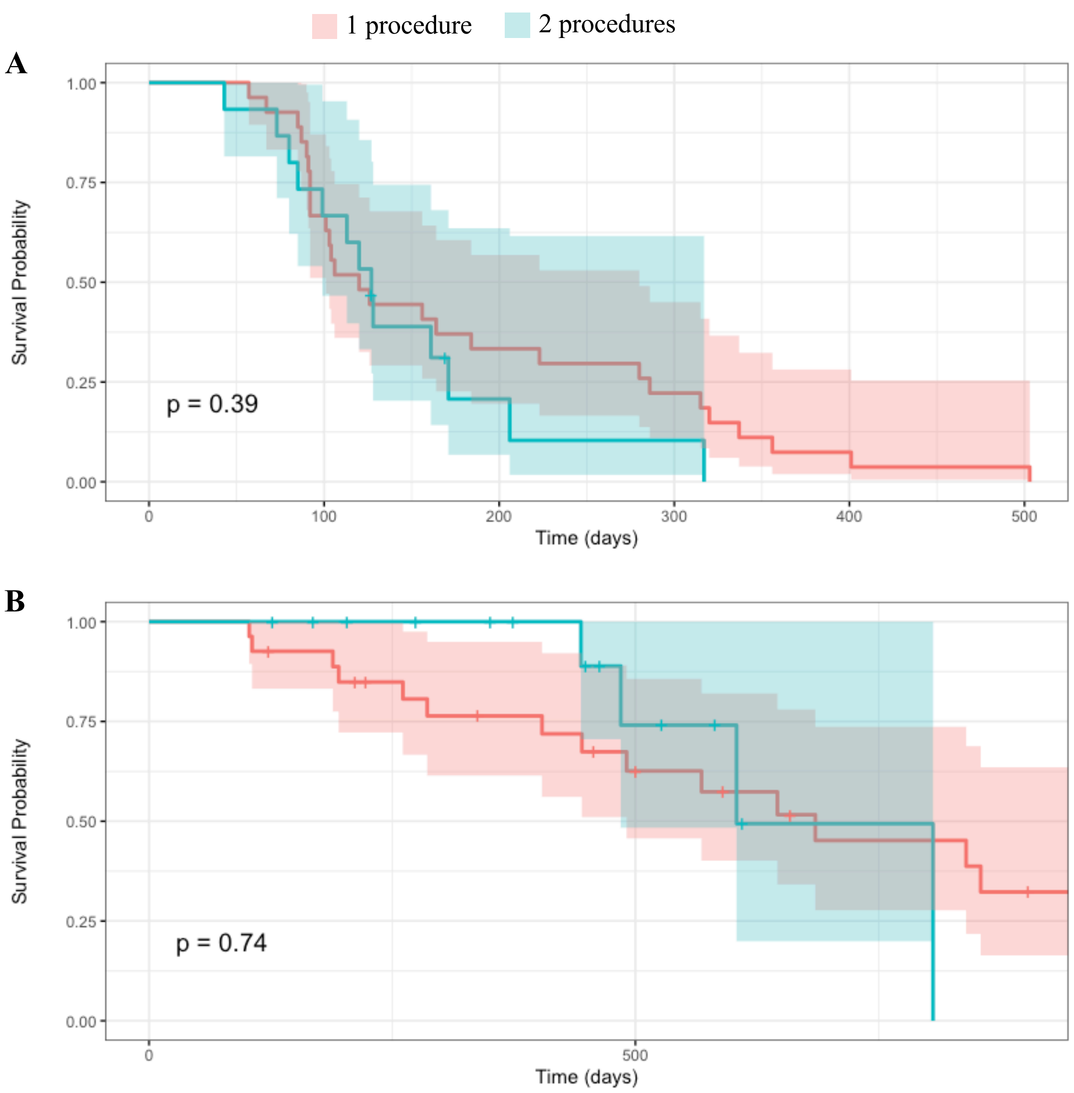

Supplemental Figure 2: Kaplan-Meier survival curves of microinvasive glaucoma surgery combined with cataract extraction in patients with normal-tension glaucoma stratified by 1 or 2 MIGS procedures. Success criteria were defined as the following: postoperative medication burden less than preoperative medication burden AND (A) postoperative intraocular pressure (IOP) reduction ≥ 30% from baseline IOP; or (B) IOP ≤ goal IOP. Goal IOP was determined to be ≥ 30% reduction from where the glaucoma specialist noted progression or the IOP at which the glaucoma specialist thought that the patient should be to prevent further progression based on clinical presentation. A failure was recorded if a patient failed to meet success criteria on at least two consecutive follow-up visits, required additional glaucoma procedures, or developed no light perception vision. Patients with no preoperative medication burden were excluded.
